# Supplementary material for: Inhibition of Mitochondrial Aconitase by Succination in Fumarate Hydratase Deficiency
Source: Cell Rep. 2013 Mar 28;3(3):689–700. doi: 10.1016/j.celrep.2013.02.013 (PMC3617368; doi:10.1016/j.celrep.2013.02.013)
Supplement: Document S1. Article plus Supplemental Information [file mmc1.pdf]

Cell Reports
Report

Cell PRESS
Open ACCESS

Inhibition of Mitochondrial Aconitase by Succination in Fumarate Hydratase Deficiency

Nicola Ternette,[1,10] Ming Yang,[2,10,*] Mahima Laroyia,[2] Mitsuhiro Kitagawa,[4] Linda O'Flaherty,[2] Kathryn Wolhulter,[2] Kaori Igarashi,[4] Kaori Saito,[4] Keiko Kato,[4] Roman Fischer,[1] Alexandre Berquand,[6] Benedikt M. Kessler,[1] Terry Lappin,[3,7] Norma Frizzell,[8] Tomoyoshi Soga,[4,5,9] Julie Adam,[2,10] and Patrick J. Pollard[2,5,9,*]
[1]Central Proteomics Facility
[2]Cancer Biology and Metabolism Group
[3]Hypoxia Biology Group
Henry Wellcome Building for Molecular Physiology, University of Oxford, Oxford OX3 7BN, UK
[4]Institute for Advanced Biosciences
[5]Oxford-Keio Metabolomics Consortium
Keio University, 246-2 Mizukami, Tsuruoka, Yamagata 997-0052, Japan
[6]Bruker Nano GmbH, Östliche Rheinbrückenstraße 49, 76187 Karlsruhe, Germany
[7]Centre for Cancer Research and Cell Biology, Queen's University, Belfast, 97 Lisburn Road, Belfast BT9 7BL, UK
[8]Department of Pharmacology, Physiology & Neuroscience, School of Medicine, University of South Carolina, Columbia, SC 29208, USA
[9]Oxford-Keio Metabolomics Consortium, Oxford OX3 7BN, UK
[10]These authors contributed equally to this work
*Correspondence: ming@well.ox.ac.uk (M.Y.), paddy@well.ox.ac.uk (P.J.P.)
http://dx.doi.org/10.1016/j.celrep.2013.02.013

SUMMARY

The gene encoding the Krebs cycle enzyme fumarate hydratase (FH) is mutated in hereditary leiomyomatosis and renal cell cancer (HLRCC). Loss of FH activity causes accumulation of intracellular fumarate, which can directly modify cysteine residues to form 2-succinocysteine through succination. We undertook a proteomic-based screen in cells and renal cysts from Fh1 (murine FH)-deficient mice and identified 94 protein succination targets. Notably, we identified the succination of three cysteine residues in mitochondrial Aconitase2 (ACO2) crucial for iron-sulfur cluster binding. We show that fumarate exerts a dose-dependent inhibition of ACO2 activity, which correlates with increased succination as determined by mass spectrometry, possibly by interfering with iron chelation. Importantly, we show that aconitase activity is impaired in FH-deficient cells. Our data provide evidence that succination, resulting from FH deficiency, targets and potentially alters the function of multiple proteins and may contribute to the dysregulated metabolism observed in HLRCC.

INTRODUCTION

Altered metabolism is a key feature and hallmark of cancer cells (Hanahan and Weinberg, 2011). How this arises, and what steps link it to oncogenesis, still eludes us. One possible answer lies with "oncometabolites," described as metabolites whose abnormal accumulation causes both metabolic and nonmetabolic (such as epigenetic) dysregulation and potential transformation to malignancy (Thompson, 2009). Fumarate hydratase (FH) has been identified as a tumor suppressor because germline loss-of-function mutations are associated with the development of hereditary leiomyomatosis and renal cell cancer (HLRCC) (Tomlinson et al., 2002). FH has roles in both the mitochondria and cytosol, catalyzing the hydration of fumarate to malate. In mitochondria, FH is a key component of the Krebs cycle, essential for cellular energy production and macromolecular biosynthesis, whereas in the cytoplasm, FH metabolizes fumarate generated from arginine synthesis and the purine nucleotide cycle (Salway, 1999; Shambaugh, 1977). Loss of FH activity results in accumulation of fumarate in cells. Elevated fumarate has been implicated in the development of FH-associated tumors through a number of pathways, e.g., by competitive inhibition of 2-oxoglutarate (2OG)-dependent oxygenases, including the hypoxia-inducible factor (HIF) hydroxylases, leading to stabilization of HIF and activation of oncogenic HIF-dependent pathways (O'Flaherty et al., 2010). However, there is increasing evidence that multiple independent pathways may have roles in FH-associated oncogenesis as a consequence of fumarate acting as an oncometabolite (Yang et al., 2012). In addition to being an allosteric inhibitor of the 2OG-dependent oxygenases similar to other oncometabolites, fumarate acts as an endogenous electrophile. It reacts spontaneously by a Michael addition reaction with free sulfhydryl groups to generate a thioether linkage with cysteine residues in proteins. This results in formation of S-(2-succino) cysteine (2SC), a process termed succination (Alderson et al., 2006). This mechanism is distinct from succinylation of cysteine in which a thioester would be formed (Zhang et al., 2011). Furthermore, 2SC immunohistochemistry is sufficiently sensitive and specific for use as a clinical biomarker of HLRCC (Bardella et al., 2011).

Significantly, succination of Kelch-like ECH-associated protein 1 (KEAP1) in FH-deficient cells leads to abrogation of its

CrossMark

interaction with the transcription factor Nuclear factor erythroid 2-related factor 2 (NRF2) and activation of the potentially oncogenic NRF2-mediated antioxidant defense pathway (Adam et al., 2011; Ooi et al., 2011). Furthermore, NRF2 activation has been shown recently to modulate cell metabolism possibly augmenting the cellular stress response (Mitsuishi et al., 2012). Elucidation of the functional consequences of KEAP1 succination prompted us to search for other 2SC targets that may contribute to the pathogenesis of FH-associated disease. Hence, we conducted a proteomic screen for 2SC in an Fh1-deficient (knockout [KO]) mouse embryonic fibroblast (MEF) cell line (O'Flaherty et al., 2010) and in murine kidney tissue and fluid where Fh1 has been deleted from the kidney tubules (Pollard et al., 2007). We identified 94 succinated proteins, including some that are succinated on functional cysteine residues. In particular, we investigated the succination of three key cysteines in the Krebs cycle enzyme, mitochondrial aconitate hydratase (Aconitase2, ACO2). We show here that fumarate-mediated succination of ACO2 impairs its enzymatic activity in a dose-dependent manner and that Fh1KO cells exhibit reduced aconitase activity. Our findings further highlight succination as a significant event that could target multiple cellular pathways in FH-associated pathogenesis.

RESULTS

Identification of 2SC Protein Targets

Previously using Fh1 MEFs, we confirmed by immunoblotting that accumulated intracellular fumarate results in high levels of 2SC in Fh1KO, but not Fh1 wild-type (WT), MEFs (Bardella et al., 2011). To detect potential 2SC targets at low abundance, we performed mitochondrial and nuclear fractionations of Fh1KO MEFs (Figure S1A). To identify 2SC targets from biological tissue, we used cystic kidneys and aspirated kidney fluid from a 30-week-old Fh1KO mouse where Fh1 is conditionally deleted in the renal tubular epithelium causing the development of hyperplastic cysts (Pollard et al., 2007). Protein extracts from mitochondrial, nuclear, and cytosolic fractions of Fh1KO MEFs and Fh1KO kidneys were separated by SDS-PAGE analyses and subjected to in-gel trypsin digestion and liquid chromatography tandem mass spectrometry (LC-MS/MS) analyses as described before (Adam et al., 2011). Combined proteomic analyses identified 4,095 proteins and 306,558 target peptide spectrum matches (PSMs) from Fh1KO MEFs (false discovery rate [FDR] 2.32%) and 3,569 proteins/226,606 PSMs from Fh1KO kidney tissue and fluid (FDR 1.96%). The MS/MS spectrum for each succination site was verified, and a total of 110 nonredundant 2SC sites were identified in 94 distinct proteins (Table 1). 2SC targets identified thus comprise proteins from diverse cellular pathways; but significantly, approximately 50% are metabolic processes (Figure S1B). Notably, ACO2, mitochondrial NFU1 iron-sulfur cluster scaffold homolog, Protein DJ-1, Peroxiredoxin-1, and Peroxiredoxin-3 are succinated on cysteine residues involved in their function (Andres-Mateos et al., 2007; Mirel et al., 1998; Tong et al., 2003; Yang et al., 2002). Also, the succination of glyceraldehyde-3-phosphate dehydrogenase (GAPDH) at C149 was confirmed as reported previously by Blatnik et al. (2008a, 2008b).

Endogenous ACO2 Is Succinated at Three Critical Cysteines in Fh1KO MEFs

To investigate the functional consequences of succination, we focused on ACO2 because of its role in the Krebs cycle, where it catalyzes the stereospecific isomerization of citrate to isocitrate via *cis*-aconitate. In particular, it is a mitochondrial oxidative stress sensor and requires an active $[Fe_4S_4]^{2+}$ cluster, bound directly by three conserved cysteine residues, for catalysis (Lloyd et al., 1999). LC-MS/MS analyses of the tryptic peptide $_{379}$VGLIGS(^{2S}C)TNSSYEDMGR$_{395}$ derived from endogenous ACO2 in Fh1KO MEFs assigned succination to C385 unambiguously (Figure 1A). The tryptic peptide spanning C448 and C451 was detected as a mixture of two isomers ($_{438}$DLGGIVLANA(^{PE}C)GP(^{2S}C)IGQWDR$_{457}$ and $_{438}$DLGGIVLANA(^{2S}C)GP(^{PE}C)IGQWDR$_{457}$) that are succinated at C451 and C448 (Figure 1B), respectively. Due to their identical mass and composition, the two succinated species could not be separated by LC-MS/MS, but measurement of resulting fragment ion masses in the MS/MS scan allowed identification of succination on both sites. Generally, we observed higher succination at C451 (~90%) compared to C448 (~10%) as determined by Mascot analysis.

Human ACO2 Is Succinated at Homologous Residues When Expressed Stably in Fh1KO MEFs

To determine if human ACO2 can be succinated on homologous cysteine residues, we transfected Fh1WT and Fh1KO MEFs with a V5-tagged *ACO2* gene. LC-MS/MS analysis following V5 immunoprecipitation confirmed succination at all three cysteine residues (C385, C448, and C451) within the active site in ACO2 expressed in only Fh1KO MEFs. To complement the mouse data, we detected succination at C448 and C451 simultaneously from the same tryptic peptide, $_{438}$DLGGIVLANA(^{2S}C)GP(^{2S}C)IGQWDRK$_{458}$ (Figure S1C).

Fumarate-Mediated Succination Reduces ACO2 Activity In Vitro

The three cysteine residues C385, 448, and 451 are crucial for iron-sulfur cluster binding in ACO2 (Figure 2A). To investigate if succination of ACO2 impairs its enzymatic activity, we preincubated pig heart ACO2 with fumarate and assayed its activity in vitro. One hour pre-exposure of ACO2 to increasing concentrations of sodium fumarate at pH 7.4 resulted in dose-dependent inhibition of its activity in the range of 1–50 mM fumarate, which parallels detection of 2SC by immunoblotting (Figure 2B). We then performed LC-MS/MS analyses of trypsin-digested ACO2 derived from assay mixtures. Succination was detected at five cysteine residues (C126, C385, C410, C451, and C592), and further, the levels of succination of the C385- and C448/C451-containing peptides increased with increasing fumarate concentration (Figures 2C, 2D, and S2A). We correlated ACO2 activity with succination in the range of 5–50 mM fumarate and obtained a negative linear correlation with succinated peptides containing ^{2S}C385 and ^{2S}C451/448 (Figure S2B). To relate the in vitro data to pathophysiological settings, we measured fumarate concentrations by capillary electrophoresis time-of-flight mass spectrometry (CE-TOFMS) (Soga et al., 2003) in FH-deficient mouse kidneys and HLRCC tumors; these were estimated to contain 1.7 ± 0.4 mM and 3.4 ± 1.2 mM fumarate, respectively

Table 1. Proteomic Screen of 2SC Targets in an Fh1-Deficient Background

Swiss-Prot Accession No.	Gene Symbol	Protein Name	Succination Site(s)	Source	PSMs	Sequence Coverage	2SC Peptide Instances
Q8BGQ7	*Aars*	alanine-tRNA ligase, cytoplasmic	C403	M(c)	302	49.9%	3
Q99KI0	*Aco2*	aconitate hydratase, mitochondrial	C385, C448, C451	M(m)	1,340	66.7%	C385(30), C451(3)
Q9R0X4	*Acot9*	acyl-coenzyme A thioesterase 9, mitochondrial	C154	K	121	57.9%	13
Q99NB1	*Acss1*	acetyl-coenzyme A synthetase 2-like, mitochondrial	C41	K	86	47.8%	6
P00329	*Adh1*	alcohol dehydrogenase 1	C83	K	508	63.7%	25
Q9WTP6	*Ak2*	adenylate kinase 2, mitochondrial	C208	K	51	53.9%	13
Q9WTP7	*Ak3*	GTP:AMP phosphotransferase, mitochondrial	C85	K	33	70.9%	4
P07724	*Alb*	serum albumin	C471	K	26,069	89.6%	501
Q9Z110	*Aldh18a1*	δ-1-pyrroline-5-carboxylate synthase	C88, C612	M(n)	1,185	77.9%	C88(10), C612(27)
P10107	*Anxa1*	annexin A1	C189, C324	M(n,c)	407	73.4%	C189(5), C324(7)
P07356	*Anxa2*	annexin A2	C223	M(n,c),K	1,001	77.0%	6
Q8K0Q5	*Arhgap18*	Rho GTPase-activating protein 18	C637	K	8	5.0%	5
Q9D0L7	*Armc10*	isoform 2 of Armadillo repeat-containing protein 10	C275	M(n)	38	57.2%	4
O55143	*Atp2a2*	sarcoplasmic/endoplasmic reticulum calcium ATPase 2	C998	M(n)	380	47.9%	8
Q91YN9	*Bag2*	BAG family molecular chaperone regulator 2	C16	M(n)	53	70.0%	2
P12658	*Calb1*	calbindin	C187	K	143	67.4%	2
Q6ZQ38	*Cand1*	cullin-associated NEDD8-dissociated protein 1	C942	M(n)	299	55.3%	6
Q8K354	*Cbr3*	carbonyl reductase (NADPH) 3	C160	K	128	83.0%	3
P80314	*Cct2*	T complex protein 1 subunit β	C535	M(n,c)	443	82.4%	5
Q9CQB5	*Cisd2*	CDGSH iron-sulfur domain-containing protein 2	C92	M(m)	26	62.2%	3
Q8BMK4	*Ckap4*	cytoskeleton-associated protein 4	C79	M(n,m), K	813	82.8%	4
P30275	*Ckmt1*	creatine kinase U-type, mitochondrial	C317	K	110	54.8%	8
Q68FD5	*Cltc*	clathrin heavy-chain 1	C870	M(n,c)	1,297	65.6%	16
A6H584	*Col6a5*	collagen α-5(VI) chain	C1974	K	267	45.8%	15
Q61656	*Ddx5*	probable ATP-dependent RNA helicase DDX5	C200	M(n)	346	62.5%	19
Q501J6-1	*Ddx17*	isoform 1 of Probable ATP-dependent RNA helicase DDX17	C191, C198	M(n)	144	56.5%	C191(1), C198(19)
Q9R0P5	*Dstn*	destrin	C23	M(c)	64	55.8%	3
Q9CQ43	*Dut*	deoxyuridine triphosphatase	C3	M(c)	65	63.6%	10
Q9JHU4	*Dync1h1*	cytoplasmic dynein 1 heavy-chain 1	C4284	M(n)	1,054	57.6%	1
Q8QZV3	*Eci1*	Dci enoyl-CoA δ isomerase 1, mitochondrial	C87	K	91	63.0%	25
P58252	*Eef2*	elongation factor 2	C41	M(c,n)	1,746	71.2%	33
Q8BGD9	*Eif4b*	eukaryotic translation initiation factor 4B	C457, C543	M(n)	231	56.0%	C457(4), C543(2)
Q8C9X6-2	*Epc1*	isoform 2 of Enhancer of polycomb homolog 1	C515	M(n)	3	4.1%	3
Q99M71-1	*Epdr1*	isoform 1 of Mammalian ependymin-related protein 1	C88	M(n)	14	37.5%	1
Q8CGC7	*Eprs*	bifunctional aminoacyl-tRNA synthetase	C744	M(c)	503	49.5%	8
Q8BTM8	*Flna*	filamin-A	C8, C574	M(n)	2,440	78.8%	C8(5), C574(27)

(*Continued on next page*)

Table 1. *Continued*

Swiss-Prot Accession No.	Gene Symbol	Protein Name	Succination Site(s)	Source	PSMs	Sequence Coverage	2SC Peptide Instances
Q80X90	*Flnb*	filamin-B	C1434, C2501	M(n), K	2,264	82.1%	C1434(25), C2501(9)
P97494	*Gclc*	glutamate-cysteine ligase catalytic subunit	C501	K	246	63.3%	6
P16858	*Gapdh*	glyceraldehyde-3-phosphate dehydrogenase	C22, C150	M(c,n)	137	62.0%	C22(13), C150 (42)
P53702	*Hccs*	cytochrome *c*-type heme lyase	C39	M(m)	117	65.4%	11
P70333	*Hnrnph2*	heterogeneous nuclear ribonucleoprotein H2	C267	M(n)	177	49.2%	15
P61979-2	*Hnrnpk*	isoform 2 of Heterogeneous nuclear ribonucleoprotein K	C132	M(n)	700	62.9%	16
Q8R081	*Hnrnpl*	heterogeneous nuclear ribonucleoprotein L	C469	M(n)	333	79.9%	8
Q9D0E1-1	*Hnrnpm*	isoform 1 of Heterogeneous nuclear ribonucleoprotein M	C26, C652	M(n)	416	80.5%	C26(3), C652(4)
P47879	*Igfbp4*	insulin-like growth factor-binding protein 4	C211	K	2	7.1%	1
Q8CAQ8-2	*Immt*	isoform 2 of Mitochondrial inner membrane protein	C172	M(m), K	14	81.8%	27
Q0GNC1-1	*Inf2*	isoform 1 of Inverted formin-2	C284	M(n)	67	35.6%	9
Q60749	*Khdrbs1*	KH domain-containing, RNA-binding, signal transduction-associated protein 1	C19	M(n), K	53	26.0%	7
P06151	*Ldha*	L-lactate dehydrogenase A chain	C84	M(c)	822	72.6%	21
P48678	*Lmna*	isoform A of Prelamin-A\C	C572	M(n), K	201	60.6%	4
Q3UMR5	*Mcu*	calcium uniporter protein, mitochondrial	C190	K	16	28.3%	4
Q9CQ65	*Mtap*	S-methyl-5′-thioadenosine phosphorylase	C130	M(c)	106	69.6%	9
Q791V5	*Mtch2*	mitochondrial carrier homolog 2	C296	K	45	36.6%	1
Q3V3R1	*Mthfd1*	monofunctional C1-tetrahydrofolate synthase, mitochondrial	C129	M(m)	1,248	82.8%	40
Q8VDD5	*Myh9*	myosin-9	C988	K	3,408	67.4%	54
Q8K2Z4-2	*Ncapd2*	isoform 2 of Condensin complex subunit 1	C439	M(n)	176	42.8%	12
Q3UYV9	*Ncbp1*	nuclear cap-binding protein subunit 1	C44	M(n)	64	31.5%	9
Q9QZ23	*Nfu1*	NFU1 iron-sulfur cluster scaffold homolog, mitochondrial	C213	M(m)	47	43.5%	3
Q9CRB2	*Nhp2*	H\ACA ribonucleoprotein complex subunit 2	C18	M(n)	22	66.0%	7
Q99LX0	*Park7*	protein DJ-1	C106	K	50	89.9%	2
Q8BKZ9	*Pdhx*	pyruvate dehydrogenase protein X component, mitochondrial	C170	M(m)	25	31.5%	1
Q5SUR0	*Pfas*	phosphoribosylformylglycinamidine synthase	C1055	M(c)	128	32.8%	5
Q9ESW8	*Pgpep1*	pyroglutamyl-peptidase 1	C108	K	11	34.4%	6
Q80UU9	*Pgrmc2*	membrane-associated progesterone receptor component 2	C75	M(n,m)	34	38.2%	5
Q61753	*Phgdh*	D-3-phosphoglycerate dehydrogenase	C369	M(c)	191	45.6%	35
Q9Z0T6	*Pkdrej*	polycystic kidney disease and receptor for egg jelly-related protein	C136	M(c)	3	1.3%	1
Q99K51	*Pls3*	plastin-3	C104	K	407	75.1%	5
P35700	*Prdx1*	peroxiredoxin-1	C173	K	236	88.4%	26
P20108	*Prdx3*	thioredoxin-dependent peroxide reductase, mitochondrial	C230	K	47	64.6%	7
P99029-1	*Prdx5*	peroxiredoxin-5, mitochondrial	C96	M(m)	215	74.1%	21
Q9R0Q7	*Ptges3*	prostaglandin E synthase 3	C58	M(c)	54	44.3%	13

(*Continued on next page*)

Table 1. *Continued*

Swiss-Prot Accession No.	Gene Symbol	Protein Name	Succination Site(s)	Source	PSMs	Sequence Coverage	2SC Peptide Instances
Q8VI36	*Pxn*	paxillin	C535, C538	K	64	61.9%	5
Q64012	*Raly*	RNA-binding protein Raly	C255	M(n), K	58	64.2%	11
Q60973	*Rbbp7*	histone-binding protein RBBP7	C116	M(n)	69	74.8%	2
Q8BG51-3	*Rhot1*	isoform 3 of Mitochondrial Rho GTPase 1	C535	K	17	14.3%	1
P62717	*Rpl18a*	60S ribosomal protein L18a	C22, C109	M(n,m,c)	287	58.0%	C22(10) C109(30)
P47955	*Rplp1*	60S acidic ribosomal protein P1	C61	M(n,m,c)	111	67.5%	30
Q91YQ5	*Rpn1*	dolichyl-diphosphooligosaccharide protein glycosyltransferase subunit 1	C478	M(m,n)	605	68.8%	11
D3YXK2	*Safb*	scaffold attachment factor B	C81	M(n)	82	35.5%	4
O70456	*Sfn*	14-3-3 protein σ	C38	K	29	61.3%	2
Q8VEM8	*Slc25a3*	phosphate carrier protein, mitochondrial	C71	K	288	61.1%	12
Q9CYN2	*Spcs2*	signal peptidase complex subunit 2	C26	M(m,n), K	143	61.1%	73
Q62266	*Sprr1a*	cornifin-A	C41, C120	K	9	43.8%	C41(2), C120(4)
Q64674	*Srm*	spermidine synthase	C89	M(c)	58	44.0%	3
Q921F2	*Tardbp*	TAR DNA-binding protein 43	C50	M(n)	16	63.8%	8
Q9R099	*Tbl2*	transducin β-like protein 2	C43	M(n)	78	59.5%	3
O08784	*Tcof1*	treacle protein	C580	M(n,c)	67	29.5%	12
Q61029-1	*Tmpo*	isoform β of Lamina-associated polypeptide 2, isoforms β\δ\ε\γ	C362	M(n)	11	61.5%	13
P17751	*Tpi1*	triosephosphate isomerase	C71, C77	K	233	81.5%	13
P21107-2	*Tpm3*	isoform 2 of Tropomyosin α-3 chain	C233	M(n)	30	71.8%	11
Q9Z1Q9	*Vars*	valyl-tRNA synthetase	C41	M(n), K	328	53.4%	2
Q60930	*Vdac2*	voltage-dependent anion-selective channel protein 2	C77, C228	M(m), K	489	65.7%	C77(2), C228(1)
Q60931	*Vdac3*	voltage-dependent anion-selective channel protein 3	C8, C65	M(m), K	365	66.1%	C8(3), C65(13)
Q62468	*Vil1*	villin-1	C134	K	268	60.2%	23

2SC targets identified from Fh1KO MEFs and mouse kidney tissue and fluid. Succinated proteins are listed alphabetically by gene symbol, with succinated cysteine residues indicated. PSM, peptide spectrum matches; M, MEFs; c, cytosolic fraction; m, mitochondrial fraction; n, nuclear fraction; K, kidney tissue or fluid. See also Figure S1.

Cell PRESS Open ACCESS

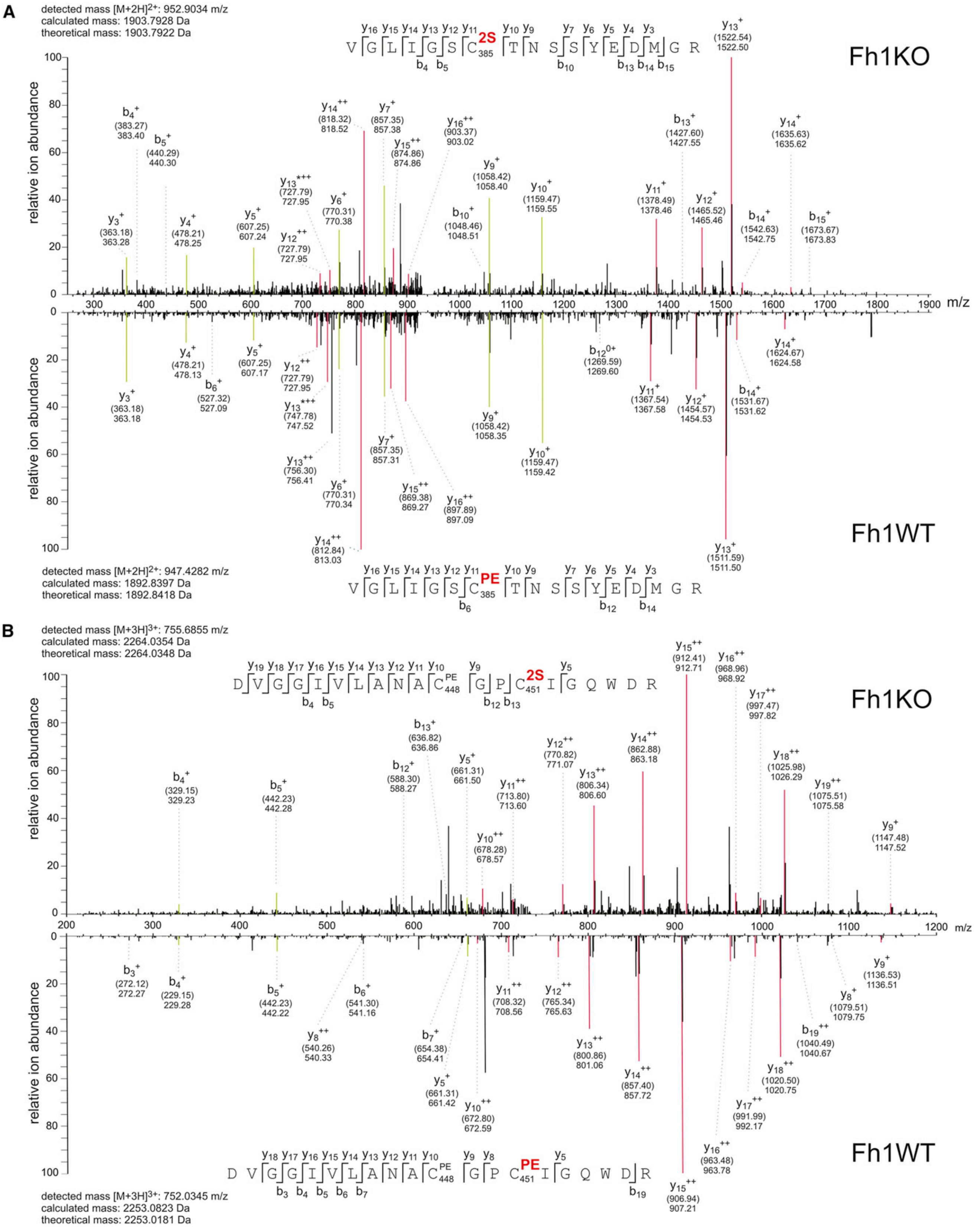

(legend on next page)

(Figures 2E and 2F). We calculated the fumarate concentration on the basis of tissue weight, but these values are likely an underestimate because the tissue is comprised of a heterogeneous population of cells, and no estimate was made of the aqueous volume of the tissue.

ACO2 Activity Is Impaired in Fh1KO MEFs

When whole-cell lysates of Fh1WT and Fh1KO MEFs were compared to determine if succination impairs endogenous ACO2 activity, Fh1KO MEFs displayed significantly reduced aconitase activity (Figure 3A). To differentiate mitochondrial and cytosolic aconitase activity (ACO2 and ACO1, respectively), we utilized two cell lines derived from the Fh1KO MEFs, reconstituted with either full-length FH (Fh1KO+FH), or FH restricted to the cytosol by deleting the mitochondrial-targeting sequence (Fh1KO+FH^{cyt}) (O'Flaherty et al., 2010). Comparison of whole-cell lysates from the four MEF cell lines showed that aconitase activity is completely restored in Fh1KO+FH cells and only partially restored in Fh1KO+FH^{cyt}, relative to that in Fh1KO cells (Figure 3A).

Previously, we showed that despite having significantly reduced total cellular fumarate compared to Fh1KO, Fh1KO+FH^{cyt} MEFs (as measured by ^{1}H-nuclear magnetic resonance spectrometry) retain abnormal mitochondria morphology and impaired respiration (O'Flaherty et al., 2010). We redetermined fumarate levels in the four cell lines by CE-TOFMS (Figure 3B) and confirmed high levels of fumarate in Fh1KO MEFs (~35 fmol/cell) and above-normal levels of fumarate in Fh1KO+FH^{cyt} (~10 fmol/cell) compared to Fh1WT (~1.5 fmol/cell) and Fh1KO+FH (~3 fmol/cell) MEFs. To relate these levels to molar concentrations, we performed cell volume measurements of the four MEF cell lines by atomic force microscopy (Schneider et al., 2004). These analyses estimated the intracellular fumarate concentrations to be ~6 mM for Fh1KO, ~0.06 mM for Fh1WT, ~0.14 mM for Fh1KO+FH, and ~1.3 mM for Fh1KO+FH^{cyt} MEFs (Figure S3). We postulated that fumarate may be accumulated in the mitochondria of Fh1KO+FH^{cyt} MEFs, and consequently, ACO2 may be succinated in these cells. Therefore, we compared ACO2 succination in the four MEF cell lines by LC-MS/MS analyses of the ^{2S}C385- and ^{2S}C451/^{2S}C448-containing tryptic peptides. Whereas no succination was detected at C385 or C451/C448 in Fh1WT or Fh1KO+FH cells, succination was detected in both Fh1KO and Fh1KO+FH^{cyt} cells with that in Fh1KO being higher (Figure 3C). Following fractionation of the four cell lines into mitochondrial versus cytoplasmic portions, we performed immunoblotting of the derived protein extracts and confirmed the presence of 2SC in both the mitochondria and cytosol of Fh1KO MEFs and also in the mitochondria of Fh1KO+FH^{cyt} MEFs (Figure S1A). Taken together, our data suggest that the partial restoration of aconitase activity in Fh1KO+FH^{cyt} MEFs may be a combined effect of functional ACO1 activity and dysfunctional ACO2 due to succination in the mitochondria.

Succination of Aconitase Causes Alterations to Metabolism in Fh1KO MEFs

To investigate if succination of aconitase might cause alteration to cellular metabolism, we first measured the levels of key Krebs cycle metabolites in Fh1WT and Fh1KO MEFs by CE-TOFMS. Consistent with FH being dysfunctional, levels of fumarate and succinate are significantly higher in Fh1KO, whereas that for malate is drastically lower (Figure 3D). Notably, we observed low levels of citrate and isocitrate in Fh1KO MEFs. We then cultured the cells in deuterium-labeled [d5]glutamine for 24 hr and analyzed them for label incorporation into these metabolites. We observed significant label incorporation in succinate (m+4) and fumarate (m+2), supporting the oxidative flux of the Krebs cycle. We also detected isocitrate m+2 but did not observe label enrichment in citrate (Figure 3E).

Some aerobic glycolytic cancer cells display altered metabolism by utilizing the glutamine-dependent reductive mechanism to produce citrate, which can be used for lipogenesis and for anaplerosis of the Krebs Cycle (Metallo et al., 2012; Mullen et al., 2012; Wise et al., 2011). This pathway uses the NADP(+)-dependent isocitrate dehydrogenase (IDH)1 and 2 to reductively carboxylate 2OG to isocitrate and is considered to occur in both the mitochondria and cytosol (Mullen et al., 2012). Our data suggest that in Fh1KO MEFs, 2OG can be converted to isocitrate by reversal of the IDH-catalyzed reaction, but isocitrate cannot be further metabolized to citrate due to impaired aconitase activity, possibly as a result of succination. Hence, succination of ACO2 may prevent Fh1KO MEFs from utilizing the reductive carboxylation pathway for citrate synthesis as adopted by some cancer cell lines. Furthermore, the absence of label in citrate suggests that both mitochondrial and cytosolic aconitase are potentially inactive in Fh1KO MEFs (Figure 3F).

DISCUSSION

Here, we report the results of a proteomic screen to identify 2SC targets in FH deficiency. We describe the succination of three cysteine residues crucial for iron-sulfur cluster binding in the active site of the Krebs cycle enzyme mitochondrial Aconitase2 (ACO2) in Fh1KO cells, which exhibit reduced aconitase activity compared to Fh1WT cells. We have demonstrated that in vitro

Figure 1. ACO2 Is Succinated on Critical Cysteine Residues in Fh1KO MEFs

(A and B) MS/MS spectra showing succination at C385 in the 379-VGLIGS(^{2S}C)TNSSYEDMGR-395 peptide (A) and at C451 in the 438-DVGGIVLANA(^{PE}C)GP(^{2S}C)IGQWDR-457 peptide (B) (upper panels) derived from endogenous ACO2 in Fh1KO MEFs. Spectra are shown in direct comparison with the originally unmodified counterpeptides that are pyridylethylated on the corresponding cysteines detected in Fh1WT cells (lower panel). Selected fragments were assigned as follows: b, N-terminal fragment ion; y, C-terminal fragment ion; *, fragment ion minus NH_3; 0, fragment ion minus H_2O; +, singly charged fragment ion; ++, doubly charged fragment ion; PE, pyridylethylated; 2S, succinated. Both theoretical mass (in brackets) and detected mass are given for each assigned fragment ion. Fragment ion mass signals that were assigned for both peptide species and contain the modified cysteine residue are highlighted in red, whereas fragments that do not comprise the modification are highlighted in green. Note that for fragment ions that include the modified cysteine, singly charged fragment ions are shifted according to the mass difference between 2S (116.01 Da) and PE (105.06) modifications by 10.95 Da, whereas doubly charged fragment signals are shifted by 5.48 Da.
See also Figure S1.

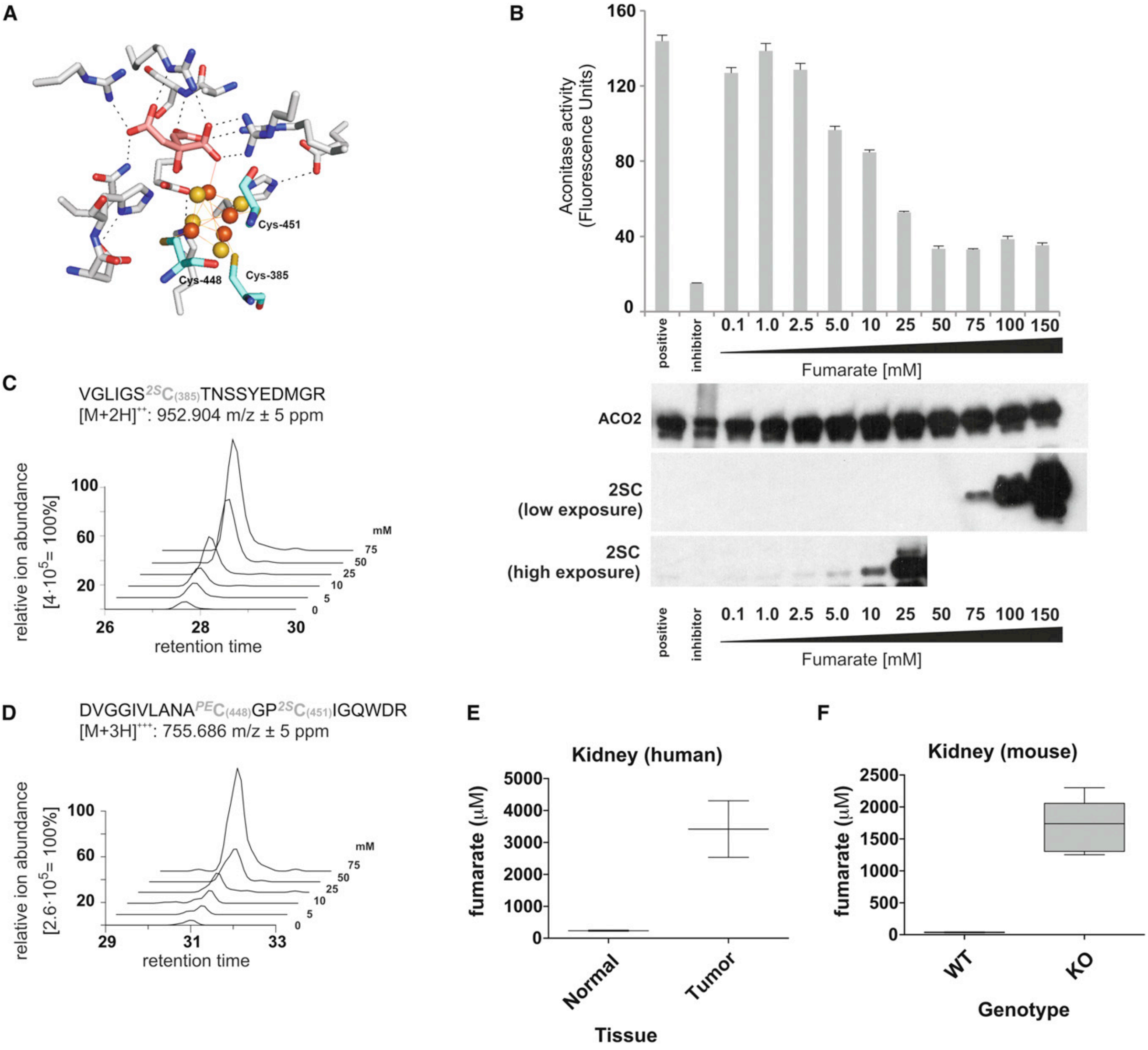

Figure 2. Fumarate-Mediated Succination Inhibits ACO2 Activity In Vitro

(A) Crystal structure showing the active site of porcine ACO2 with the substrate citrate (pink) and [4Fe-4S] cluster (orange/yellow) bound. The three iron-binding cysteine residues (C385, C447, and C451) are shown in cyan. Picture was created using PyMOL (Protein Data Bank ID code 1C96).

(B) Activity of porcine ACO2 preincubated with increasing concentrations of fumarate (0.1–150 mM) in 50 mM Tris-HCl (pH 7.4). Untreated ACO2, or ACO2 pretreated with the aconitase inhibitor oxalomalate, was used as a positive or negative control, respectively. Immunoblots for ACO2 and 2SC from the assay mixtures are displayed beneath the corresponding fumarate concentration, and two film exposures are shown for 2SC (high and low).

(C and D) Representative (one of three triplicate MS analyses) extracted ion chromatograms for the succinated tryptic peptides containing C385 (C) and C448/C451 (D) derived from porcine ACO2 purified from the aconitase assay mixture, showing the increase in succination with increasing fumarate concentration.

(E and F) CE-TOFMS analyses of fumarate concentrations in HLRCC tumors (E) and in Fh1-deficient kidneys.

All error bars indicate SEM. See also Figure S2.

inhibition of ACO2 is a direct consequence of dose-dependent fumarate-mediated succination, particularly at $\geq$5 mM fumarate, equivalent to concentrations measured in FH-deficient tissues. Because tissue samples are a heterogeneous mix of both control and FH-deleted cells, precise determination of intracellular fumarate is difficult, and the actual concentrations in these FH-deficient cells could be significantly underestimated. Fumarate concentrations in subcellular compartments, e.g., mitochondria versus cytosol, could also be variable and differentially affect local protein succination. Additionally, compared to

Cell PRESS Open ACCESS

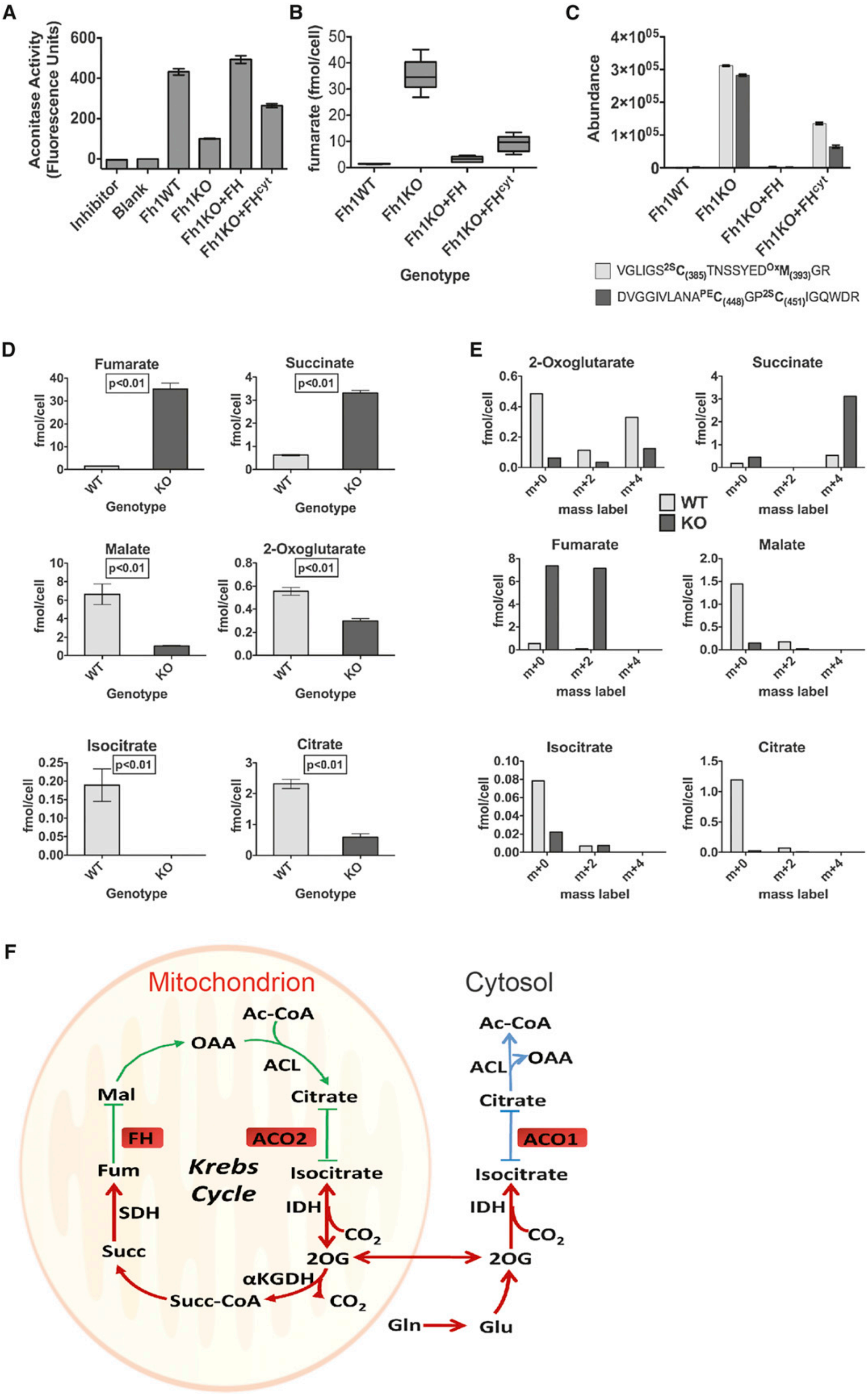

(legend on next page)

the relatively simple in vitro situation, the catalytic activity of aconitase in cells could be influenced by multiple components such as cosubstrate and cofactor availability. Interestingly, we did identify succination of proteins involved in iron-sulfur cluster assembly in our proteomic screen, potentially further hindering aconitase activity. Stable isotope tracer studies showed that Fh1KO MEFs do not utilize the reductive carboxylation mechanism for citrate synthesis, which may be a consequence of fumarate-dependent succination of ACO2, adding a further layer of complexity to the disruption of mitochondrial metabolism caused by FH deficiency.

Cytosolic Aconitase1 (ACO1) also contains three iron-sulfur-binding cysteine residues and is a bifunctional enzyme that acts either as an iron response element (IRE)-binding protein to regulate iron uptake, sequestration, and utilization or as the cytosolic aconitase, depending on iron availability (Philpott et al., 1994). Whether succination affects the IRE-binding ability of ACO1 and, by inference, iron homeostasis in FH-deficient cells is an interesting question that warrants future investigation.

2SC has been described in aging and diabetes, and its functional consequences have been reported for GAPDH and adiponectin in addition to the KEAP1/NRF2 pathway (Adam et al., 2011; Frizzell et al., 2009; Thomas et al., 2012). Our proteomic screen for 2SC targets aims to expand our current knowledge of the extent of this modification and its cellular impacts. Despite the fact that our screen is biased toward abundant proteins, it is significant that whereas proteins encompassing diverse cellular pathways are targets for succination, around half are involved in metabolism. A few proteins including the iron-sulfur cluster assembly protein NFU1 and the thioredoxin-dependent peroxide reductases are succinated on critical cysteine residues, suggesting that succination may adversely affect function in these targets. The thioether adduct generated by fumarate modifications occurs nonenzymatically and is believed to be irreversible (Alderson et al., 2006; Frizzell et al., 2011, 2012). However, it is conceivable that 2SC may influence signal transduction by targeting proteins that are cellular stress sensors such as KEAP1. Alternatively, 2SC may compete with other cysteine modifications such as S-nitrosylation and oxidation to sulfinic acid to indirectly target other cellular signaling events. Although the effects on individual proteins require closer investigations, our data provide evidence that succination is a significant posttranslational modification in FH deficiency and a potential key mechanism linking multiple pathways that may cause dysregulation of cell metabolism and contribute to oncogenesis.

EXPERIMENTAL PROCEDURES

See also Extended Experimental Procedures.

Aconitase Assay

The aconitase assay is based on the protocol described in the Aconitase Assay Kit (Cayman Chemical) with modifications. NADPH production was followed by fluorescence (excitation 340 nm; emission of 465 nm) over 45 min at 37°C. Activation of pig heart aconitase and preparation of MEF cell lysates followed the manufacturer's protocol.

Proteomics and Mass Spectrometry

Cell fractionations were performed using Qproteome Mitochondria Isolation Kit (QIAGEN), or as previously described (Adam et al., 2011). Kidney samples were homogenized and sonicated in Urea-SDS buffer (O'Flaherty et al., 2010). Protein extracts were separated by SDS-PAGE and processed for trypsin digestion and LC-MS/MS analyses as previously described (Adam et al., 2011). Database searches were performed against SwissProt (06/2011) or International Protein Index (09/2012) database using Mascot (Perkins et al., 1999) or CPFP 1.3.0 (Trudgian et al., 2010). For label-free quantitation of succinated peptides, samples were analyzed in three technical replicates. Relative quantitation was performed using Progenesis LC-MS v.4.0. Correlation analysis was performed using GraphPad Prism v.5 assuming Gaussian populations (Pearson) calculating two-tailed p values with a confidence interval of 95%. Tissue and cell samples for metabolite analysis by CE-TOFMS were prepared as described before (Adam et al., 2011; Soga et al., 2006, 2009).

Mice and Human Tissue Samples

All procedures were conducted in line with American Association for Cancer Research guidelines and performed under UK Home Office regulations after approval by the Local Ethical Review Process at Oxford University. Anonymized human tumor and normal samples were collected with full ethical approval (MREC 05/Q1605/66) as approved by the Oxford Centre for Histopathology Research.

SUPPLEMENTAL INFORMATION

Supplemental Information includes three figures and Extended Experimental Procedures and can be found with this article online at http://dx.doi.org/10.1016/j.celrep.2013.02.013.

LICENSING INFORMATION

This is an open-access article distributed under the terms of the Creative Commons Attribution License, which permits unrestricted use, distribution, and reproduction in any medium, provided the original author and source are credited.

Figure 3. Aconitase Activity Is Reduced in Fh1KO MEFs

(A) Aconitase activities of Fh1WT, Fh1KO, Fh1KO+FH, and Fh1KO+FH^{cyt} MEF cell lysates normalized to cell number. The aconitase inhibitor oxalomalate was used as a negative control.

(B) CE-TOFMS analyses of fumarate concentration in the four MEF cell lines.

(C) Extent of succination of ACO2 at C385 and C451/448 in the four MEF cell lines determined by measuring abundance of the relevant peptides by LC-MS/MS.

(D) CE-TOFMS analyses confirmed significant differences between Fh1WT and KO MEFs in the levels of the key Krebs cycle metabolites fumarate, succinate, malate, 2OG, isocitrate, and citrate.

(E) Mass isotopomer analysis of key Krebs cycle metabolites in Fh1WT and Fh1KO MEFs cultured with [d5]glutamine for 24 hr.

(F) Schematic of glutamine metabolism by the Krebs cycle in Fh1KO MEFs. Abbreviations are as follows: Ac-CoA, acetyl coenzyme A; ACL, ATP citrate lyase; ACO1 and ACO2, Aconitases 1 and 2; FH, fumarate hydratase; FUM, fumarate; Gln, glutamine; Glu, glutamate; IDH, isocitrate dehydrogenase; Mal, malate; OAA, oxaloacetate; Succ, succinate; Succ-CoA, succinyl coenzyme A; SDH, succinate dehydrogenase; 2OG, 2-oxoglutarate; αKGDH, α-ketoglutarate dehydrogenase.

All error bars indicate SEM. See also Figure S3.

ACKNOWLEDGMENTS

We thank Climent Casals-Pascual and David Trudgian for their helpful advice on data analysis. This work was funded by the Wellcome Trust (WT091112MA to P.J.P.), Cancer Research UK (A13349, A14607, and A12027 to P.J.P.), a Grant-in-Aid for scientific research on Innovative Areas, Japan (No. 22134007 to T.S.), and the Yamagata Prefectural Government and City of Tsuruoka. The European Research Council has provided financial support under the European Community's Seventh Framework Programme (FP7/2007-2013)/ERC grant agreement no. 310837 to P.J.P. T.S. is a founder of Human Metabolome Technologies, and A.B. is an applications scientist employed by Bruker.

Received: October 28, 2012
Revised: January 28, 2013
Accepted: February 11, 2013
Published: March 14, 2013

REFERENCES

Adam, J., Hatipoglu, E., O'Flaherty, L., Ternette, N., Sahgal, N., Lockstone, H., Baban, D., Nye, E., Stamp, G.W., Wolhuter, K., et al. (2011). Renal cyst formation in Fh1-deficient mice is independent of the Hif/Phd pathway: roles for fumarate in KEAP1 succination and Nrf2 signaling. Cancer Cell *20*, 524–537.

Alderson, N.L., Wang, Y., Blatnik, M., Frizzell, N., Walla, M.D., Lyons, T.J., Alt, N., Carson, J.A., Nagai, R., Thorpe, S.R., and Baynes, J.W. (2006). S-(2-Succinyl)cysteine: a novel chemical modification of tissue proteins by a Krebs cycle intermediate. Arch. Biochem. Biophys. *450*, 1–8.

Andres-Mateos, E., Perier, C., Zhang, L., Blanchard-Fillion, B., Greco, T.M., Thomas, B., Ko, H.S., Sasaki, M., Ischiropoulos, H., Przedborski, S., et al. (2007). DJ-1 gene deletion reveals that DJ-1 is an atypical peroxiredoxin-like peroxidase. Proc. Natl. Acad. Sci. USA *104*, 14807–14812.

Bardella, C., El-Bahrawy, M., Frizzell, N., Adam, J., Ternette, N., Hatipoglu, E., Howarth, K., O'Flaherty, L., Roberts, I., Turner, G., et al. (2011). Aberrant succination of proteins in fumarate hydratase-deficient mice and HLRCC patients is a robust biomarker of mutation status. J. Pathol. *225*, 4–11.

Blatnik, M., Frizzell, N., Thorpe, S.R., and Baynes, J.W. (2008a). Inactivation of glyceraldehyde-3-phosphate dehydrogenase by fumarate in diabetes: formation of S-(2-succinyl)cysteine, a novel chemical modification of protein and possible biomarker of mitochondrial stress. Diabetes *57*, 41–49.

Blatnik, M., Thorpe, S.R., and Baynes, J.W. (2008b). Succination of proteins by fumarate: mechanism of inactivation of glyceraldehyde-3-phosphate dehydrogenase in diabetes. Ann. N Y Acad. Sci. *1126*, 272–275.

Frizzell, N., Rajesh, M., Jepson, M.J., Nagai, R., Carson, J.A., Thorpe, S.R., and Baynes, J.W. (2009). Succination of thiol groups in adipose tissue proteins in diabetes: succination inhibits polymerization and secretion of adiponectin. J. Biol. Chem. *284*, 25772–25781.

Frizzell, N., Lima, M., and Baynes, J.W. (2011). Succination of proteins in diabetes. Free Radic. Res. *45*, 101–109.

Frizzell, N., Thomas, S.A., Carson, J.A., and Baynes, J.W. (2012). Mitochondrial stress causes increased succination of proteins in adipocytes in response to glucotoxicity. Biochem. J. *445*, 247–254.

Hanahan, D., and Weinberg, R.A. (2011). Hallmarks of cancer: the next generation. Cell *144*, 646–674.

Lloyd, S.J., Lauble, H., Prasad, G.S., and Stout, C.D. (1999). The mechanism of aconitase: 1.8 A resolution crystal structure of the S642a:citrate complex. Protein Sci. *8*, 2655–2662.

Metallo, C.M., Gameiro, P.A., Bell, E.L., Mattaini, K.R., Yang, J., Hiller, K., Jewell, C.M., Johnson, Z.R., Irvine, D.J., Guarente, L., et al. (2012). Reductive glutamine metabolism by IDH1 mediates lipogenesis under hypoxia. Nature *481*, 380–384.

Mirel, D.B., Marder, K., Graziano, J., Freyer, G., Zhao, Q., Mayeux, R., and Wilhelmsen, K.C. (1998). Characterization of the human mitochondrial aconitase gene (ACO2). Gene *213*, 205–218.

Mitsuishi, Y., Taguchi, K., Kawatani, Y., Shibata, T., Nukiwa, T., Aburatani, H., Yamamoto, M., and Motohashi, H. (2012). Nrf2 redirects glucose and glutamine into anabolic pathways in metabolic reprogramming. Cancer Cell *22*, 66–79.

Mullen, A.R., Wheaton, W.W., Jin, E.S., Chen, P.H., Sullivan, L.B., Cheng, T., Yang, Y., Linehan, W.M., Chandel, N.S., and DeBerardinis, R.J. (2012). Reductive carboxylation supports growth in tumour cells with defective mitochondria. Nature *481*, 385–388.

O'Flaherty, L., Adam, J., Heather, L.C., Zhdanov, A.V., Chung, Y.L., Miranda, M.X., Croft, J., Olpin, S., Clarke, K., Pugh, C.W., et al. (2010). Dysregulation of hypoxia pathways in fumarate hydratase-deficient cells is independent of defective mitochondrial metabolism. Hum. Mol. Genet. *19*, 3844–3851.

Ooi, A., Wong, J.C., Petillo, D., Roossien, D., Perrier-Trudova, V., Whitten, D., Min, B.W., Tan, M.H., Zhang, Z., Yang, X.J., et al. (2011). An antioxidant response phenotype shared between hereditary and sporadic type 2 papillary renal cell carcinoma. Cancer Cell *20*, 511–523.

Perkins, D.N., Pappin, D.J., Creasy, D.M., and Cottrell, J.S. (1999). Probability-based protein identification by searching sequence databases using mass spectrometry data. Electrophoresis *20*, 3551–3567.

Philpott, C.C., Klausner, R.D., and Rouault, T.A. (1994). The bifunctional iron-responsive element binding protein/cytosolic aconitase: the role of active-site residues in ligand binding and regulation. Proc. Natl. Acad. Sci. USA *91*, 7321–7325.

Pollard, P.J., Spencer-Dene, B., Shukla, D., Howarth, K., Nye, E., El-Bahrawy, M., Deheragoda, M., Joannou, M., McDonald, S., Martin, A., et al. (2007). Targeted inactivation of fh1 causes proliferative renal cyst development and activation of the hypoxia pathway. Cancer Cell *11*, 311–319.

Salway, J.G. (1999). Metabolism at a Glance, Second Edition (Malden, MA: Blackwell Science).

Schneider, S.W., Matzke, R., Radmacher, M., and Oberleithner, H. (2004). Shape and volume of living aldosterone-sensitive cells imaged with the atomic force microscope. Methods Mol. Biol. *242*, 255–279.

Shambaugh, G.E., 3rd. (1977). Urea biosynthesis I. The urea cycle and relationships to the citric acid cycle. Am. J. Clin. Nutr. *30*, 2083–2087.

Soga, T., Ohashi, Y., Ueno, Y., Naraoka, H., Tomita, M., and Nishioka, T. (2003). Quantitative metabolome analysis using capillary electrophoresis mass spectrometry. J. Proteome Res. *2*, 488–494.

Soga, T., Baran, R., Suematsu, M., Ueno, Y., Ikeda, S., Sakurakawa, T., Kakazu, Y., Ishikawa, T., Robert, M., Nishioka, T., and Tomita, M. (2006). Differential metabolomics reveals ophthalmic acid as an oxidative stress biomarker indicating hepatic glutathione consumption. J. Biol. Chem. *281*, 16768–16776.

Soga, T., Igarashi, K., Ito, C., Mizobuchi, K., Zimmermann, H.P., and Tomita, M. (2009). Metabolomic profiling of anionic metabolites by capillary electrophoresis mass spectrometry. Anal. Chem. *81*, 6165–6174.

Thomas, S.A., Storey, K.B., Baynes, J.W., and Frizzell, N. (2012). Tissue distribution of S-(2-succino)cysteine (2SC), a biomarker of mitochondrial stress in obesity and diabetes. Obesity (Silver Spring) *20*, 263–269.

Thompson, C.B. (2009). Metabolic enzymes as oncogenes or tumor suppressors. N. Engl. J. Med. *360*, 813–815.

Tomlinson, I.P., Alam, N.A., Rowan, A.J., Barclay, E., Jaeger, E.E., Kelsell, D., Leigh, I., Gorman, P., Lamlum, H., Rahman, S., et al.; Multiple Leiomyoma Consortium. (2002). Germline mutations in FH predispose to dominantly inherited uterine fibroids, skin leiomyomata and papillary renal cell cancer. Nat. Genet. *30*, 406–410.

Tong, W.H., Jameson, G.N., Huynh, B.H., and Rouault, T.A. (2003). Subcellular compartmentalization of human Nfu, an iron-sulfur cluster scaffold protein, and its ability to assemble a [4Fe-4S] cluster. Proc. Natl. Acad. Sci. USA *100*, 9762–9767.

Trudgian, D.C., Thomas, B., McGowan, S.J., Kessler, B.M., Salek, M., and Acuto, O. (2010). CPFP: a central proteomics facilities pipeline. Bioinformatics *26*, 1131–1132.

Wise, D.R., Ward, P.S., Shay, J.E., Cross, J.R., Gruber, J.J., Sachdeva, U.M., Platt, J.M., DeMatteo, R.G., Simon, M.C., and Thompson, C.B. (2011). Hypoxia promotes isocitrate dehydrogenase-dependent carboxylation of α-ketoglutarate to citrate to support cell growth and viability. Proc. Natl. Acad. Sci. USA *108*, 19611–19616.

Yang, K.S., Kang, S.W., Woo, H.A., Hwang, S.C., Chae, H.Z., Kim, K., and Rhee, S.G. (2002). Inactivation of human peroxiredoxin I during catalysis as the result of the oxidation of the catalytic site cysteine to cysteine-sulfinic acid. J. Biol. Chem. *277*, 38029–38036.

Yang, M., Soga, T., Pollard, P.J., and Adam, J. (2012). The emerging role of fumarate as an oncometabolite. Front. Oncol. *2*, 85.

Zhang, Z., Tan, M., Xie, Z., Dai, L., Chen, Y., and Zhao, Y. (2011). Identification of lysine succinylation as a new post-translational modification. Nat. Chem. Biol. *7*, 58–63.

Supplemental Information

EXTENDED EXPERIMENTAL PROCEDURES

Cell Lines and Immunoprecipitation

Four mouse embryonic fibroblasts (MEFs) cell lines were used: Fh1+/+ (Fh1WT), Fh1−/− (Fh1KO), and isogenic Fh1KO MEFs, reconstituted with either full-length FH (Fh1KO+FH), or cytosolic-restricted FH by deleting the mitochondrial targeting sequence (Fh1KO+FH^{cyt}) (O'Flaherty et al., 2010). Human ACO2 gene was amplified from HEK293T cDNA libraries by PCR and ligated into pEF1/V5-HisA vector (Invitrogen). Transfection used FuGene®6 (Roche) following the manufacturer's protocol. Cell culture was performed as previously described (O'Flaherty et al., 2010). Immunoprecipitation of V5-tagged ACO2 was performed according to the manufacturer's protocol using V5 agarose beads (Invitrogen).

Immunoblotting and Antibodies

Primary antibodies used were V5 (Invitrogen), FH (Autogen Bioclear), COXIV (Cell Signaling Technology), ACO1 (Cell Signaling Technology), ACO2 (Cell Signaling Technology) and 2SC (Nagai et al., 2007) and TUBB (Abcam). Immunoblotting was performed as previously described (Adam et al., 2011; O'Flaherty et al., 2010).

Mice

Tissue and aspirated fluid from cystic kidneys were obtained from $Fh1^{fl/fl}$ Ksp-cre (Fh1KO) as described previously (Adam et al., 2011; Pollard et al., 2007).

Aconitase Assay

Pig heart aconitase (39.03 μg/ml) (Cayman) was activated by incubation at 4°C for 1 hr in 475 mM Tris-Cl, pH 7.4, containing 1.6 mM cysteine hydrochloride and 17 mM ferrous ammonium sulfate and assayed in a final total volume of 205 μl containing 256 mM Tris-Cl, pH 7.4; 0.4 mM cysteine hydrochloride; 4.25 μM ferrous ammonium sulfate; 0.5 mM sodium citrate; 0.25 mM $NADP^+$ and 3.75 U of isocitrate dehydrogenase. For in vitro succination porcine ACO2 was incubated with sodium fumarate (0-150 mM final concentration) 15 min at 4°C prior to activation. Aconitase activity was determined by the rate of NADPH production by fluorescence (excitation 340 nm; emission of 465 nm) over 45 min 37°C (Halangk and Kunz, 1991) in a Safire 2 plate reader (Tecan). Cell lysates for aconitase assay were prepared by harvesting the MEFs at 4°C in Homogenization Buffer (50 mM Tris-HCl, pH 7.4, 0.1 mM sodium citrate), followed by sonication and centrifugation at 14,000 rpm for 10 min at 4°C to clear the lysate.

Mass Spectrometry

For proteomics identification of 2SC targets, protein extracts were separated by SDS-PAGE and excised sequentially into 6X 1cm-long gel slides before cutting to small pieces for further processing. hACO2 immunoprecipitated from Fh1WT and Fh1KO MEFs and porcine ACO2 derived from aconitase assay mixtures were separated by SDS-PAGE and gel bands corresponding to the size of ACO2 were excised. All gel slices were subjected to reductive alkylation and in-gel trypsin digestion as previously described (Adam et al., 2011).

Peptides were separated on an Acquity nano UPLC system (Waters) supplemented with a 25 cm C18 column, 1.7 μm particle size (Waters) using a linear gradient from 3% buffer A (0.1% formic acid in water) to 40% buffer B (0.1% formic acid in acetonitrile) at a flow rate of 250 nl/min (approx. 7000 psi) from 0 to 60 or 90 min according to the complexity of the sample. Peptides were ionized and introduced to an LTQ Orbitrap Velos tandem mass spectrometer (Thermo Scientific) using an electrospray ionization (ESI) source. Collision induced dissociation (CID) was induced on the twenty most abundant ions per full MS scan using an isolation width of 1.5 Da. All fragmented precursor ions were actively excluded from repeated MS/MS analysis for 15 s. Raw data was converted to Mascot generic files using msconvert (Kessner et al., 2008). Searches were performed using the SwissProt database (06/2011) with MASCOT (Perkins et al., 1999) and CPFP 1.3.0 (Trudgian et al., 2010) with the following settings: Variable modifications: 2-succinyl (cysteine, +116.01 Da), pyridylethyl (cysteine, +105.06 Da), oxidation (methionine, +15.00 Da), peptide tolerance: ± 10 ppm, fragment tolerance: ± 0.5 Da.

Atomic Force Microscopy

Atomic force microscopy was performed as previously described (Binnig et al., 1986; Oberleithner et al., 2003; Schneider et al., 2004; Schneider et al., 1997) using a Bioscope Catalyst instrument (Bruker Nano GmbH). MEFs were plated overnight at low confluence in standard DMEM. Measurements were made using ScanAsyst-Fluid probes in PFQNM mode, regular SW (drive amplitude 300 nm) but special workspace (drive frequency 0.5 kHz). A minimum of 5 locations were scanned for each cell line and at least 1 high resolution image was captured.

D5 Glutamine-2,3,3,4,4 Labeling of Cells and CE-TOFMS

Cells were cultured in standard DMEM for 12 hr and then in medium containing 4 mM [d5]-Glutamine (Glutamine-2,3,3,4,4) for 24 hr prior to processing for isotope incorporation and metabolite analysis. Anionic and cationic metabolite profiling were performed as described previously (Soga et al., 2009; Soga et al., 2003). The automatic recalibration of each acquired spectrum was performed

against reference masses of reference standards ([^{13}C isotopic ion of protonated methanol dimer (2MeOH^{+}H)]$^{+}$, m/z 66.0632) and ([Hexakis (2,2- difluorothoxy)phosphazene +H] $^{+}$, m/z 622.0290) and exact mass data were acquired as outlined in (Soga et al., 2006). Essentially all other details are as described previously (Soga et al., 2009).

SUPPLEMENTAL REFERENCES

Binnig, G., Quate, C.F., and Gerber, C. (1986). Atomic force microscope. Phys. Rev. Lett. *56*, 930–933.

Halangk, W., and Kunz, W.S. (1991). Use of NAD(P)H and flavoprotein fluorescence signals to characterize the redox state of pyridine nucleotides in epididymal bull spermatozoa. Biochim. Biophys. Acta *1056*, 273–278.

Kessner, D., Chambers, M., Burke, R., Agus, D., and Mallick, P. (2008). ProteoWizard: open source software for rapid proteomics tools development. Bioinformatics *24*, 2534–2536.

Nagai, R., Brock, J.W., Blatnik, M., Baatz, J.E., Bethard, J., Walla, M.D., Thorpe, S.R., Baynes, J.W., and Frizzell, N. (2007). Succination of protein thiols during adipocyte maturation: a biomarker of mitochondrial stress. J. Biol. Chem. *282*, 34219–34228.

Oberleithner, H., Schneider, S.W., Albermann, L., Hillebrand, U., Ludwig, T., Riethmüller, C., Shahin, V., Schäfer, C., and Schillers, H. (2003). Endothelial cell swelling by aldosterone. J. Membr. Biol. *196*, 163–172.

Schneider, S.W., Yano, Y., Sumpio, B.E., Jena, B.P., Geibel, J.P., Gekle, M., and Oberleithner, H. (1997). Rapid aldosterone-induced cell volume increase of endothelial cells measured by the atomic force microscope. Cell Biol. Int. *21*, 759–768.

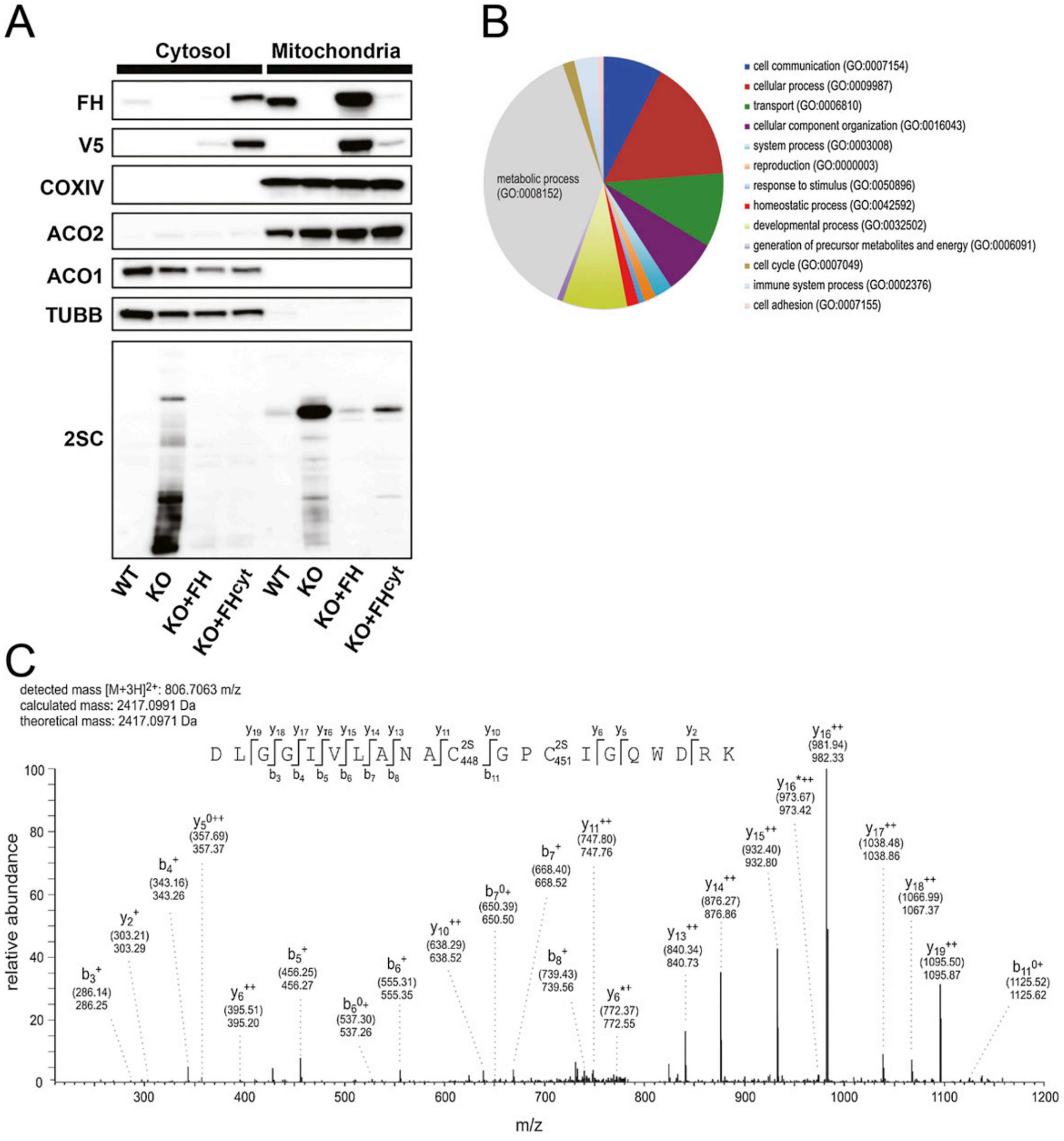

Figure S1. Succination of Protein Targets in Fh1KO MEFs, Related to Table 1 and Figure 1

(A) Mitochondrial fraction of Fh1WT, Fh1KO, Fh1+FH and Fh1+FHcyt MEFs with immunoblotting for respective subcellular markers, FH and 2SC.

(B) Pie chart showing the diversity and relative proportions of biological processes encompassing proteins identified as 2SC targets. Chart created using PANTHER (Protein Analysis Through Evolutionary Relationships) (http://www.pantherdb.org/).

(C) MS/MS spectrum showing succination at both C448 and C451 in the $_{438}$-DVGGIVLANA(^{2S}C)GP(^{2S}C)IGQWDR-457 peptide derived from human ACO2 expressed stably in Fh1KO MEFs. Selected fragments were assigned as follows: b: N-terminal fragment ion; y: C-terminal fragment ion; *: fragment ion minus NH_3; 0: fragment ion minus H_2O; +: singly charged fragment ion and ++: doubly charged fragment ion; PE: pyridylethylated; 2S:succinated. Both theoretical mass (in brackets) and detected mass are given for each assigned fragment ion.

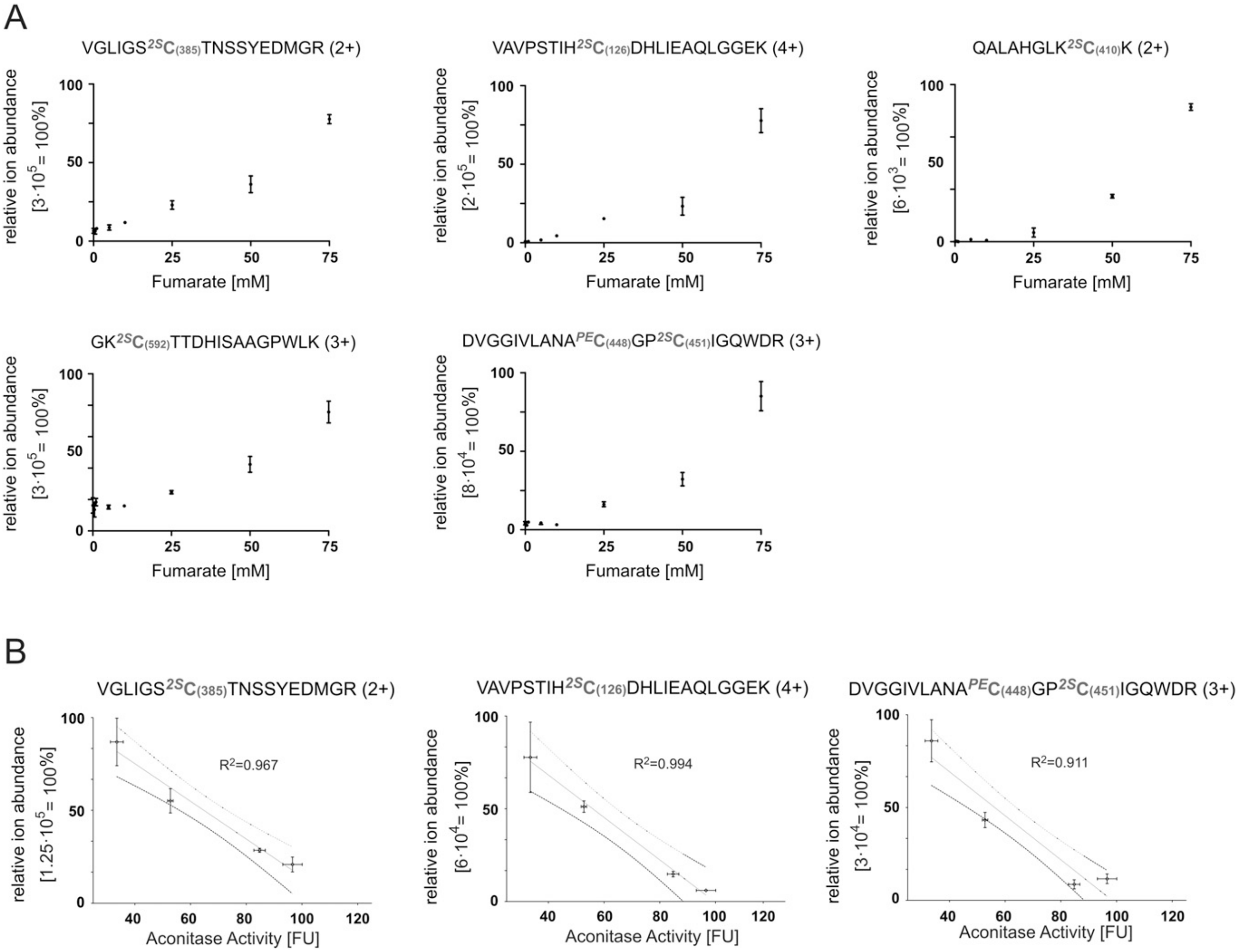

Figure S2. Increasing Concentrations of Fumarate Correlate with Increasing Succination at Residues C385, 448, C451, and C126 and Decreasing ACO2 Activity, Related to Figure 2

(A) Relative ion abundance of the most intense charge state (in brackets) of all detected distinct succinated peptides of porcine ACO2 in the activity assay samples. Modified residues are indicated in bold grey including the aa position in ACO2. 2S: 2-succination, PE: pyridylethylation.

(B) Analysis of correlation between the ion abundance of indicated succinated peptides and measured aconitase activity in the dynamic range of both activity assay and sensitivity of MS instrument (5-50mM Fumarate).

All error bars indicate SEM.

Cell PRESS
Open ACCESS

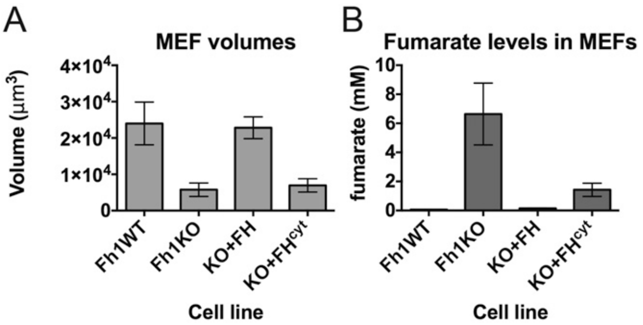

Figure S3. Determination of Fumarate Concentration in Fh1 MEFs by CE-TOFMS Analyses and Atomic Force Microscopy, Related to Figure 3
(A) Estimated cell volumes of Fh1 MEFs measured by atomic force microscopy.
(B) Estimated fumarate concentrations based on quantitation of metabolites by CE-TOFMS analyses and atomic force microscopic measurement of cell volumes. All error bars indicate SEM.
